# Calibration of High Heat Flux Sensors at NIST

**DOI:** 10.6028/jres.102.032

**Published:** 1997

**Authors:** A. V. Murthy, B. K. Tsai, C. E. Gibson

**Affiliations:** Aero-Tech Inc. Hampton, VA 23666; National Institute of Standards and Technology, Gaithersburg, MD 20899-0001

**Keywords:** absolute technique, calibration, heat flux, irradiance, radiation, sensor, transfer technique, uncertainties

## Abstract

An ongoing program at the National Institute of Standards and Technology (NIST) is aimed at improving and standardizing heat-flux sensor calibration methods. The current calibration needs of U.S. science and industry exceed the current NIST capability of 40 kW/m^2^ irradiance. In achieving this goal, as well as meeting lower-level non-radiative heat flux calibration needs of science and industry, three different types of calibration facilities currently are under development at NIST: convection, conduction, and radiation. This paper describes the research activities associated with the NIST Radiation Calibration Facility. Two different techniques, transfer and absolute, are presented. The transfer calibration technique employs a transfer standard calibrated with reference to a radiometric standard for calibrating the sensors using a graphite tube blackbody. Plans for an absolute calibration facility include the use of a spherical blackbody and a cooled aperture and sensor-housing assembly to calibrate the sensors in a low convective environment.

## 1. Introduction

A program to develop competence in characterizing heat flux sensors is in progress at the National Institute of Standards and Technology. This program includes the development or upgrading of separate radiative, convective, and conduction heat transfer facilities in which a variety of sensors can be calibrated. This paper deals with work being carried out in the Optical Technology Division (OTD), Physics Laboratory (PL), to fulfill the needs for the radiative calibration portion of the program.

The OTD is involved in establishing a standard radiation calibration technique and in upgrading the Radiation Calibration Facility (RCF) at NIST. Previous heat-flux characterization studies in the division used a transfer calibration method and covered up to 40 kW/m^2^ irradiance. Current needs of U.S. industry and laboratories, however, exceed this level [1]. For example, in the area of fire research and testing, extant test methods call for irradiance levels up to 100 kW/m^2^ [2].

Towards meeting this objective, several options are under consideration to update the RCF. This paper reviews the present capabilities and assesses different options to extend these capabilities to characterize heat flux sensors up to 100 kW/m^2^.

In a parallel effort, the Building and Fire Research Laboratory (BFRL), NIST, is developing separate convective and conductive heat transfer facilities that will permit calibration of sensors used to measure non-radiative modes of heat transfer. The convective facility consists of a 1.0 cm by 30 cm channel through which a tightly-controlled flow of air is maintained. An electrically heated wall produces heat fluxes up to 5 kW/m^2^. The conduction facility is designed to transfer over 50 kW/m^2^ across a 1 mm helium gap, with the radiation being kept below 2 % of the total heat flow. These nonradiative calibrations will not be discussed further in this paper.

## 2. Calibration Methods

The methods used to characterize heat flux sensors fall into two categories: transfer and absolute. Central to the basic calibration in both methods is the radiant source used to provide the required level of irradiance at the sensor location.

### 2.1 Transfer Calibration

The transfer method [3] presently in use at NIST uses a two-step procedure with the calibration traceable to a radiometric standard. This method, described in detail in Sec. 3, does not use the measured temperatures for heat flux calculations, but the measured signal output of the sensor is directly related to the heat flux measured by a transfer standard radiometer. A high temperature graphite tube blackbody is used as a transfer source for broadband calibration of the sensor with reference to a transfer standard radiometer. Departure of the transfer source from blackbody behavior has little effect on the calibration, since the transfer standard is itself calibrated against a primary radiometric standard.

### 2.2 Absolute Calibration Method

The absolute calibration of heat flux sensors is conceptually simple. The irradiance at the sensor surface can be calculated knowing the blackbody temperature, enclosure geometry, and the surface emissivities. No reference to a primary or transfer standard radiometer is necessary. This approach, which has been successfully employed in Sweden by Olsson [4], is described in detail in Sec. 4.

## 3. NIST Transfer Technique

### 3.1 Method

The present approach for calibrating heat flux sensors at NIST employs a transfer technique in a two-step calibration. In the first step, a cavity-type transfer standard is characterized against a primary radiometric standard using a laser beam as a transfer source. Next, the heat flux sensor to be calibrated is characterized with reference to the transfer standard using a graphite tube radiator transfer source.

The primary standard [5] used in the first step is a quantum efficiency detector (QED) which is an absolute radiometric standard in the visible range (360 nm to 800 nm). Complete conversion of the incident photon flux is achieved by three silicon photodiodes, geometrically arranged to give a total of five absorbing surfaces in the light path. The silicon photodiodes are connected in parallel so that the output current of the detector is equal to the sum of the individual currents. The accuracy is within 0.1 % of the maximum attainable quantum efficiency [6]. The output current *I*_qed_ of the detector is related to the input power *P* and wavelength of the incident radiation by

$$I_{\text{qed}}=k\;P\gamma$$ (1)

where *k* = (10^9^/1239.5) A·W^−1^·m^−1^. It must be noted that the linear measurement range is 1 nW to 50 W with no applied bias voltage, and 1 W to 2 mW with bias.

The transfer standard [7] is an electrically calibrated radiometer (ECR). This is an absolute cavity radiometer operating on the principle of equivalence between thermodynamic heating and electrical heating. Due to the fact that the temperature, electrical voltage, and current can be measured with high degree of accuracy, it is generally considered that the ECR measurements are absolute. However, with the presence of factors such as long term drift, uncertainties in the cavity heating, etc., it is desirable that the ECR be characterized with respect to a primary standard, as described above, when higher accuracy is desired. At NIST, the practice is to calibrate the ECR against the primary standard (QED) using an argon or krypton ion laser with single line operation.

**Fig. 1:**
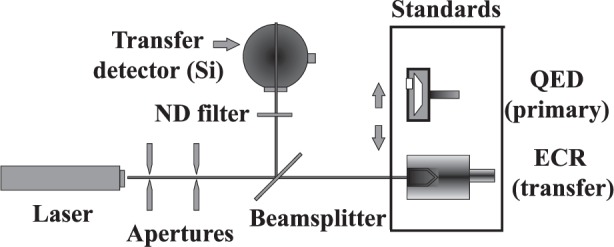
Schematic diagram of NIST technique for transfer standard calibration (step 1). The quantum efficiency detector (QED) is considered an absolute radiometer. While the electrically calibrated radiometer is considered a transfer standard.

**Fig. 2:**
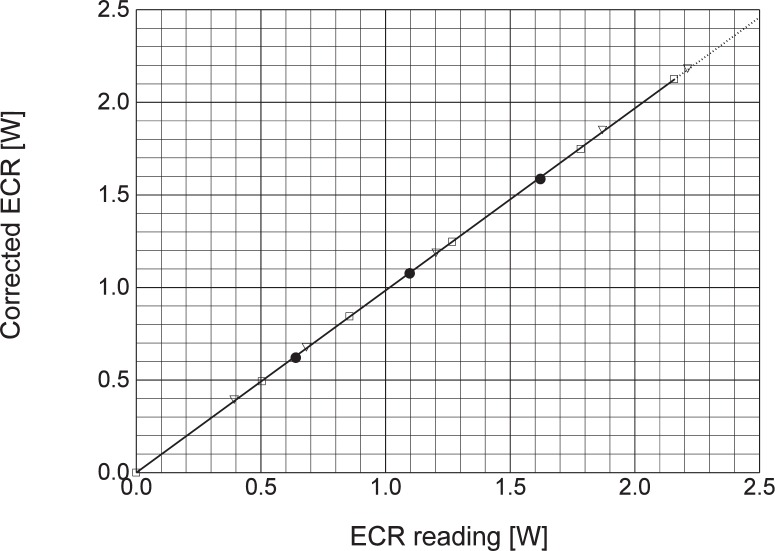
Plot showing the calibration of the ECR transfer standard using krypton layer as a transfer source.

Figure 1 shows a schematic of the experimental setup used in calibrating the transfer standard. The laser beam, after being defined by the apertures, passes through a plate beamsplitter. The transmitted beam is incident on the primary standard or the transfer standard. A silicon detector (SiD) with a suitable integrating sphere and/or neutral density filters is used to measure the reflected beam to establish the intermediate calibration with the QED. First, the QED is positioned in front of the transmitted beam. The QED calibration is transferred to the silicon detector using very low power within the operating range of the QED. Next, the transfer standard (ECR) is positioned in front of the transmitted beam. Readings of the silicon detector and the ECR are taken over the full range of laser power of interest to the calibration. The calibration of the silicon detector obtained in the first step is considered valid over the full power of the transfer standard to be calibrated (ECR), which is five to six orders of magnitude greater than the QED. This is possible due to the well-established linearity of the silicon detector in this calibration range of interest [8]. Figure 2 shows a recent calibration of the ECR using a krypton laser beam (647.1 nm). The responsivity of the primary standard QED becomes nonlinear at this wavelength and cannot be calculated using Eq. (1). Hence, the responsivity of the QED was determined experimentally in the wavelength range 600 nm to 700 nm in the Visible/Near Infrared Spectral Comparator Facility [9].

**Fig. 3:**
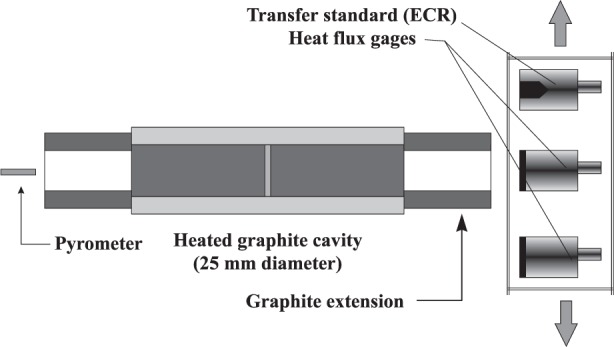
Schematic diagram depicting step 2 of the NIST technique for heat flux gage calibration.

**Fig. 4:**
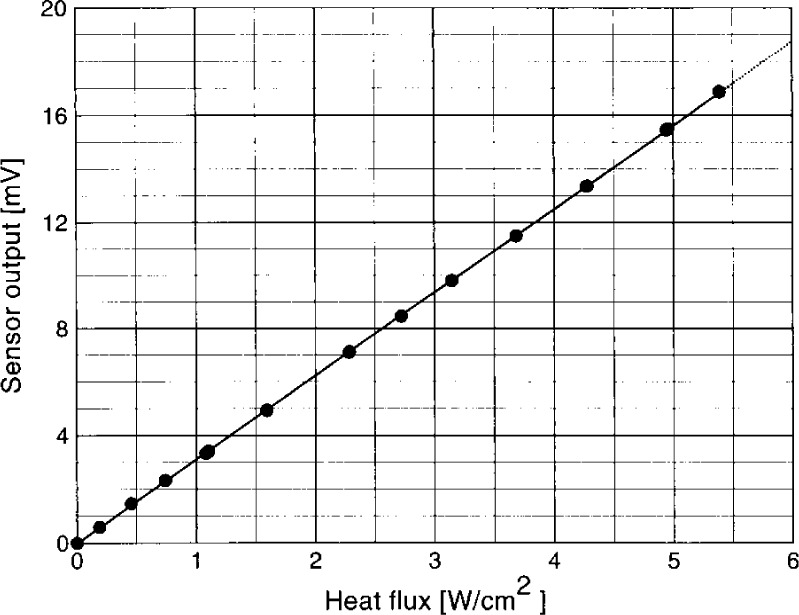
Plot showing the typical calibration of a Schmidt-Boelter type heat flux sensor in the 25 mm variable temperature blackbody (VTBB).

The transfer of the ECR calibration to the heat flux sensor is performed using a variable temperature black-body (VTBB) transfer source. The VTBB is an electrically heated graphite tube (25 mm diameter, 3 mm thick) with a center partition. The temperature of the cavity is controlled by a pyrometer sensing radiation from one end of the tube. The radiation from the other end provides heat flux for the gages and the transfer standard. The cavity geometry is symmetric about the center partition. This helps in maintaining nearly equal amounts of argon gas flow used for purging the VTBB on both sides of the partition. Figure 3 shows a schematic of the experimental arrangement. The heat flux sensors and the transfer standard are mounted at a fixed distance from the exit of the cavity. After heating the VTBB to the desired temperature, it is positioned in front of the gages as well as the transfer standard, and the signal outputs are recorded. The temperature of the VTBB is within 0.1 K of the set temperature over long periods of time, thus permitting sequential measurement of the gage and transfer standard outputs. It must be noted that the absolute value of the blackbody temperature is not critical to the test as long as it is stable over the test duration. Figure 4 shows typical calibration data for a heat flux sensor measured using the VTBB. Attendant uncertainties are discussed in Sec. 3.2, which follows.

### 3.2 Estimates of Data Uncertainty

In this type of transfer test, uncertainties are introduced at two stages. (Throughout the remainder of this paper, all uncertainty values are given as a relative expanded uncertainty with a coverage factor of *k* = 2. This is equivalent to an uncertainty of 2 standard deviations about the mean of a normalized Gaussian distribution.) The factors affecting the uncertainties during calibration of the transfer standard (ECR) with the primary standard (QED) using the laser arise mainly from fluctuations in laser power during the measurement. To account for this effect, and considering the long time constant of the ECR, the detector (QED and SiD) and ECR readings were taken over a period of 60 s to 100 s and the mean values were determined. A conservative estimate for the uncertainty due to drift in laser power during this time period is 0.1 % of the mean value when operating at less than 0.5 W, and much lower at higher powers. The total uncertainty in the calibration of the transfer standard is estimated to be 1.1 % (*k* = 2) of the mean value.

Second, uncertainties occur while transferring the ECR calibration to the heat flux gages using VTBB for broadband testing. The temperature of the VTBB during the test, measured by the pyrometer, was steady within about 0.1 K of the set value. The outputs of the ECR and gages were recorded over a period of 60 s to 100 s. The standard deviation of the mean of the measured values was less than 0.1 % of the mean at high heat flux levels above 5 kW/m^2^, and 0.2 % at lower heat flux levels. The uncertainty in transferring the calibration from the transfer standard to the heat flux sensors is estimated to be 1 % of the mean value. Combining this uncertainty with the uncertainty in the transfer standard calibration in step 1, the total uncertainty in the calibration of the heat flux sensors is about 1.5 % (*k* = 2) of the mean value.

### 3.3 Merits and Limitations

To date, the transfer technique described above has been used for calibrating sensors [10] up to about 50 kW/m^2^. The use of an absolute radiometric standard (QED) for reference reduces the uncertainties associated with the radiation from the VTBB aperture. It is only necessary to position the transfer standard and the sensor at the same distance from the VTBB. This can be done easily using a spacer block or a positioning mechanism. The errors due to positioning will be small (< 0.4 %) and can be estimated easily. Other extraneous effects peculiar to the experimental setup will cause similar small or measurable changes in the irradiance levels on both the transfer standard and the sensor. Hence, the calibration of the sensor will be relatively unaffected by unknown or other experimental factors which are difficult to determine.

The transfer technique is presently being used in the “open mode,” that is, with no enclosure containing the radiation from the blackbody. This is advantageous in accommodating a larger variety of sensor configurations. It is necessary that the distribution of the heat flux across the sensor surface be uniform. The errors due to placing the sensor at different locations from the aperture are discussed later Sections. One of the disadvantages of the open mode is the cooling of the sensor surface due to free convection effects. Care must be taken to minimize the convection effects or to account for them properly. Another problem that is of particular concern to the NIST technique is the effect of argon gas flow used to purge the graphite VTBB. The gas flow exiting at the open end of the VTBB extension in the vicinity of the sensor can change the heat flux at the sensor surface. An assessment of this effect obtained from a recent test is described in a later section.

The transfer technique can also be used in a “closed mode,” that is, with a suitable cooled enclosure containing the blackbody and the sensor or transfer standard. The cooled enclosure minimizes the convection heat loss at the surface. (As discussed in Sec. 4, the experimental setup can be also used for direct, absolute calibration of the sensor.) However, closed mode operation can limit the size and type of the gage holder, and proper accounting for the reflected radiation within the enclosure is necessary.

The present method assumes that the first step of the transfer standard calibration, which is carried out at one wavelength, is valid over the broadband radiation of the VTBB. Past measurements [11] of the cavity blackness at five different wavelengths of a similar ECR have shown the variation in reflectance is about 1 % to 2 %. It is planned to verify the spectral response of the ECR by calibration at two or more wavelengths. So far, the practice has been to calibrate at only one wavelength and to assume that the calibration is valid over the broadband radiation of the VTBB.

The NIST technique, so far in use up to about 50 kW/m^2^ irradiance level, can be extended to higher irradiance levels (≈ 100 kW/m^2^) with the use of a suitable transfer standard and transfer source. However, to realize such high irradiance levels at the sensor surface with the present VTBB, it is necessary to use either a shorter extension or no extension piece on one side of the VTBB to position the sensor close to the mouth of the radiating cavity. Furthermore, during transfer calibration, the main consideration is stability of the VTBB temperature rather than its absolute value. The ECR used as transfer standard has a time constant of 6 s (63.2 %), and it is desirable to wait for about 60 s after a change in the test conditions. Hence, stable temperature of the VTBB over the test duration is a requirement. The time constants of the gages are generally much smaller, less than 100 ms (63.2 %), in the range up to 10 kW/m^2^.

## 4. Swedish Absolute Calibration Technique

**Fig. 5:**
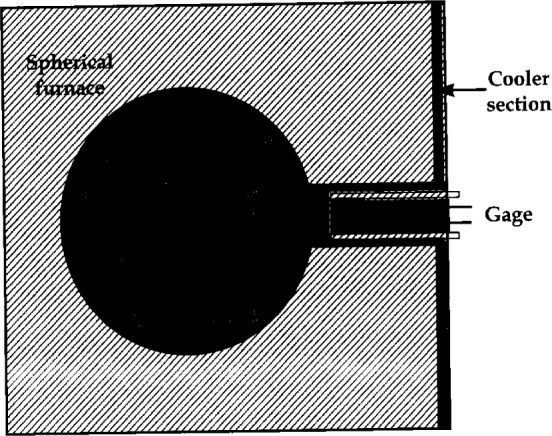
Schematic diagram depicting the Swedish blackbody setup for the absolute calibration of heat flux gages.

This technique uses a spherical blackbody and a water cooled aperture and sensor-housing assembly (Fig. 5). The “cooler assembly” and the associated sensor mounting enclosure were specially designed to minimize background radiation. The design also substantially reduces the convection effects at the sensor surface. The irradiance at the sensor surface was calculated by considering the radiation balance in an enclosure. The initial version [4] of the facility was suitable for calibration between 10 kW/m^2^ to 60 kW/m^2^. The facility was later improved to enable calibration in the range 20 kW/m^2^ to 100 kW/m^2^ [12].

The Olsson technique has received considerable attention because of substantially reduced convection effects and the ability to calibrate up to 100 kW/m^2^ with a source temperature of about 1473 K, close to source temperatures of interest in fire research. The facility is designed to locate the sensor in two positions: close to the aperture for high heat flux levels (6 kW/m^2^ to 100 kW/m^2^), and away from the aperture for the low flux levels (2 kW/m^2^ to 30 kW/m^2^). The total relative uncertainty in calibration is ± 2.8 % of the calculated radiation. This uncertainty estimate is obtained by conservatively adding the individual uncertainties.

Presently, the calibration results from the Swedish facility rely entirely on the blackbody environment of the furnace. The apparent emissivity of the furnace is calculated to be 0.997. Hence, the radiation from the aperture will be close to blackbody radiation. The determination of the heat flux at the sensor surface by Planck’s radiation law eliminates the need for using a transfer standard.

## 5. Discussion

The following discussion is aimed towards developing thermal radiation facilities at NIST to calibrate sensors up to 100 kW/m^2^. Because the two types of heat flux sensor calibration methods discussed above, the NIST and Swedish, have features that are complementary, it is planned to develop facilities at NIST to conduct direct comparative studies between the two techniques. Options to upgrade the present NIST facility and also procurement of other readily available equipment, including a spherical blackbody, are now under consideration to meet the goals of the heat flux program. The following sections present some of the major issues involved in reaching this goal.

### 5.1 Furnace Type and Emissivity

The VTBB (Fig. 3) has a graphite tube furnace of 25.4 mm diameter and a heated zone of 14.1 cm on either side of the center partition, with a length-to-diameter ratio of about 5.5. The calculated effective normal emissivity for this configuration varies from 0.997 to 0.999 for furnace wall emissivities of 0.7 to 0.9, assuming a uniform wall temperature distribution. The corresponding values for the Swedish spherical furnace are between 0.995 and 0.999. However, it must be noted that the temperature near the aperture drops in both furnaces due to heat transfer to cooler surfaces or the ambient environment. More complex calculations are necessary to assess this effect.

The 25 mm VTBB at NIST has an apparent emissivity of 0.99 and is being mainly used as a variable temperature transfer source. By reducing the temperature gradients along the length of the cavity with a modified design, the emissivity can be increased, with the result that the radiation from the aperture is nearer to black-body radiation. Consequently, a modified VTBB has the potential for use as an absolute as well as a transfer source.

**Fig. 6:**
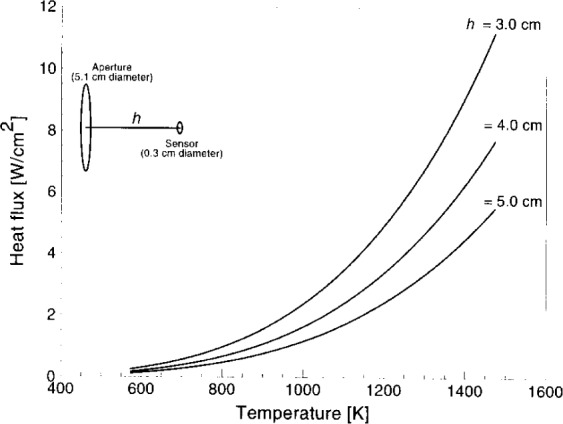
Plot showing the calculated heat flux levels at the sensor location for the 23 cm spherical blackbody for the absolute calibration of heat flux gages.

Design calculations have been completed for a 230 mm diameter spherical blackbody with a 50 mm diameter radiating aperture, an emissivity of 0.999 ± 0.0005, and a maximum temperature of 1473 K. These characteristics are close to the characteristics of the Swedish furnace described in [12]. Figure 6 shows the calculated heat flux levels obtainable with the spherical furnace for different locations of the sensor and furnace temperature. Consequently, with a suitable design of additional accessories, it seems promising to obtain the required heat flux levels at a temperature of 1473 K, which is an appropriate source temperature for many fire-research applications.

### 5.2 Realization of Heat Flux Levels

The VTBB has a standard uncooled extension, 16.1 cm long, on either side of the heated zone (Fig. 3). In this configuration, the argon gas flow used for purging flows nearly in equal proportion on both sides of the furnace partition. The extension provides a zone of laminar flow for the argon gas. This is the standard configuration used in most of the studies. However, the standard extension limits the maximum heat flux level at the gage location to about 10 kW/m^2^.

**Fig. 7:**
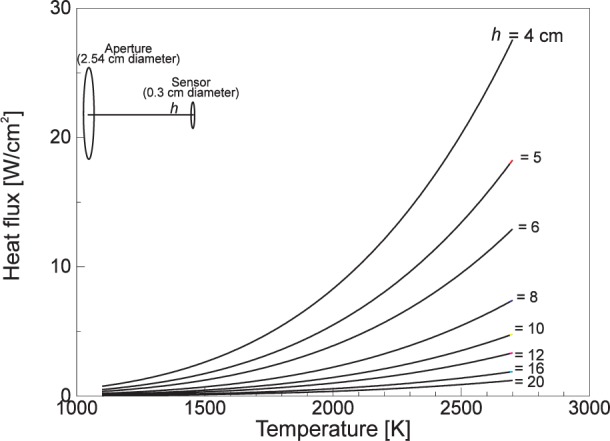
Plot showing the calculated heat flux levels at the sensor location for the 25 mm variable temperature blackbody (VTBB).

**Fig. 8:**
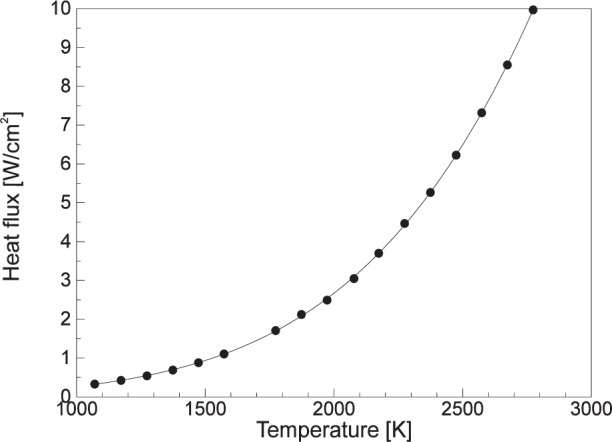
Plot for the measured heat flux from a Gardon gage located at 5.4 cm from the variable temperature blackbody exit (VTBB). The curve represents a 4th degree polynomial fit to the measured data.

To realize higher heat flux levels, it is necessary to locate the gage closer to the cavity aperture. Figure 7 shows the calculated heat flux levels for different sensor locations and furnace temperature for the 25 mm VTBB. A shorter extension piece (8.5 cm long) in place of the standard was tried and found successful in realization of heat flux levels of about 50 kW/m^2^. The short extension piece decreases the length of the laminar flow zone and creates unequal flow of argon on two sides of the partition. Realization of much higher heat flux levels, up to 100 kW/m^2^, was possible in a recent exploratory study by operating the VTBB without any extension piece, and placing the gage at a distance of about 5.4 cm from the exit of the heated zone. Figure 8 shows the measured heat flux levels by a Gardon gage with increasing VTBB temperature. It must be noted that without an extension piece on one side of the VTBB, the argon gas flow on two sides of the furnace partition will be unequal. However, its effect on irradiance at the sensor surface during a transfer calibration is likely to be secondary. Nevertheless, the curtailment of the laminar flow zone reduces the effective life of the graphite blackbody. To minimize this effect, modifications to the present VTBB test set up are necessary for use at 100 kW/m^2^ irradiance. In all these studies, the VTBB was operated up to a maximum temperature of about 2773 K.

### 5.3 Convection Heat Loss

**Fig. 9:**
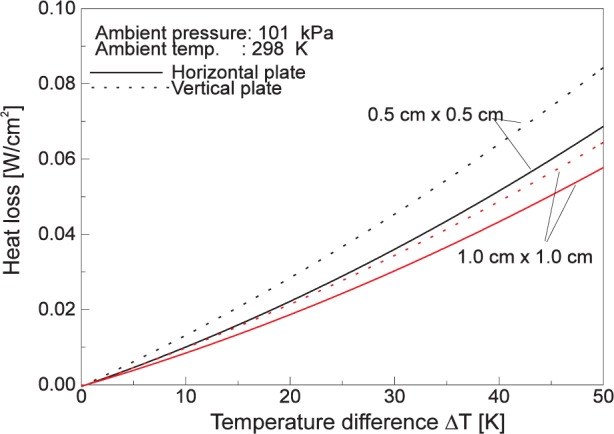
Plot showing the estimated convection loss from heated horizontal and vertical plates in air.

Unless the heat flux gages are calibrated in vacuum, the free convection flow generated at the sensor surface due to its higher temperature than the surroundings carries away heat from the sensor surface. This leads to a net reduction of the heat flux measured by the sensor. While a detailed calculation can be involved, some estimates of the convective heat loss can be obtained by considering simple configurations of the sensor surface [13]. Figure 9 shows the results for two sizes of heated plates in horizontal and vertical orientations, respectively. The results are valid for air at ambient conditions and up to a maximum temperature rise of 50 K. For the 25 mm VTBB, the gas in the vicinity of the sensor surface can be a mixture of argon and air. However, the calculations by Olsson [4] show that the difference in convective heat transfer between argon and air is not significant. Generally, the convection losses become higher with decreasing sensor area. If the gage surface temperature increase is limited to 50 K, it may be possible to limit the convective loss to less than about 1 kW/m^2^. However, these calculations should be performed for specific test conditions to confirm the convective losses are within the desired accuracy, or if necessary, to correct for convection losses.

While considering the heat loss due to convection, additional consideration of the convection effects with specific reference to calibrating in the VTBB setup is necessary. The temperature rises of the ECR cavity and the sensor surface preferably should be of the same magnitude to ensure that errors due to convection losses are about the same.

### 5.4 Purge Gas Flow Effects

The VTBB and other graphite blackbodies operating at high temperatures use some inert gas, like argon, for purging the cavity. Depending on the location of the gage, the low velocity argon jet exiting from the black-body will introduce additional effects. In a steady flow situation, the centerline velocity of a narrow axisymmetric jet [14] decreases of as *x*^−1^, where *x* is the distance from the jet exit. Therefore, the farther the gage is situated away from the blackbody cavity, the smaller the jet impingement velocity and the associated stagnation point heat transfer effect, and the more dominant are the free convection effects. However, the irradiance at the sensor decreases much faster (as a function of the square of the distance) and realization of high heat flux levels is not possible.

With the need for locating gages close to the black-body cavity exit to realize high heat flux levels, an understanding of the jet flow effect on the gage output is necessary. It must be noted that the jet momentum can be small and can become unstable at a short distance from the exit. For the 25 mm VTBB, the total flow rate of argon gas is about 0.8 × 10^−6^ m^3^/s. Assuming the flow is divided equally on both sides and uniform distribution of flow across the tube cross section, the average velocity at the exit is about 0.08 m/s. This low velocity jet can become unstable and cause unsteady heat transfer at the gage surface.

**Fig. 10:**
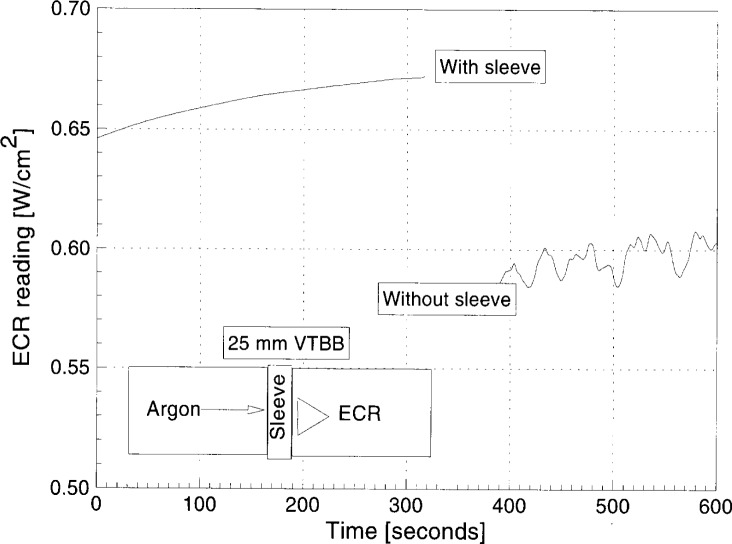
Plot showing argon jet flow effects on irradiance at ECR location, with and without sleeve, for 25 mm variable temperature blackbody (VTBB). Total argon flow rate = 0.03 m^3^/h.

Figure 10 shows the results of an experiment to study the effect of argon gas flow in the 25 mm VTBB. In this experiment, the ECR described earlier was placed at a distance of 1.27 cm from the exit. A removable hollow spacer block (1.27 cm long) was used to measure the effects of confining the argon gas flow. Despite the long time constant of the ECR, the strong effect of the argon jet exiting from the aperture on the ECR output is seen. However, with the spacer block in position, the jet flow was almost blocked and the unsteadiness in the ECR output is almost absent. Therefore, the low gas flow environment is beneficial in minimizing the stagnation point flow heat transfer effects on calibration. The increase in heat flux level with the spacer block at the sensor surface is due to confined radiation in the enclosure. The gradual increase in the heat flux with time is expected to be due to the heating of the graphite extension which is not cooled.

The results of this experiment show the ways to minimize the argon flow effects in the present setup. The case considered is at a low heat flux of about 6 kW/m^2^ with the blackbody operating at a temperature of 1573 K. At higher flux levels, the signal levels are much higher and the effect of argon flow will be smaller. This experiment suggests the following two approaches to modify the present 25 mm VTBB setup for use at higher heat fluxes up to 100 kW/m^2^.

- Provide a water cooled extension in placed of the uncooled graphite extension.
- Locate the gages and the ECR inside the extension thus confining the argon flow passage.

The fluctuation in the output observed without the spacer block will be much larger if a more sensitive gage like a Schmidt-Boelter gage is being tested. More detailed studies of the argon flow are necessary to further quantify the heat transfer at the gage surface.

### 5.5 Configuration Factors and Heat Flux Distribution

**Fig. 11:**
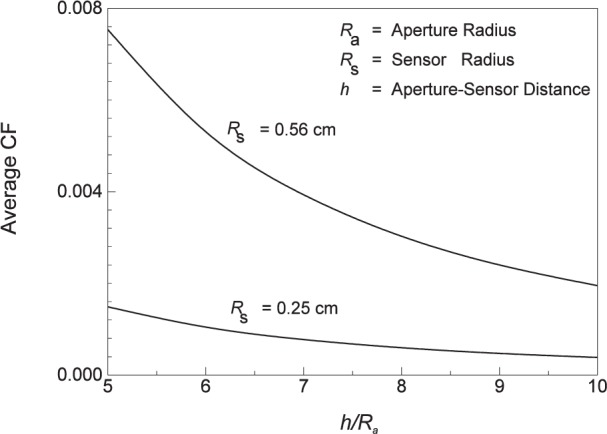
Plot of the variation of configuration factor *CF* with sensor location for the NIST 25 mm variable temperature blackbody (VTBB).

The amount of energy radiated from the blackbody that is received at the gage location depends on the aperture radius *R*_a_, sensor radius *R*_s_, and the distance *h* between them. Since the sensor has a finite area, the distribution of the energy across the radius is maximum at the center and decreases towards the edge. It is usual to define a configuration factor *CF* to calculate the total average energy received at the sensor location. The quantity *CF* represents the fraction of the energy radiated by the blackbody that is received at the sensor, and is a function of geometry only (*R*_a_, *R*_s_ and *h*). Figure 11 shows the calculated variation of the average *CF* for two different sensor radii and different distances from the aperture. The larger sensor radius of 0.56 cm corresponds to the transfer standard (ECR) aperture. As expected, *CF* decreases with distance from the aperture.

In addition to the average *CF*, how the view angle varies for each elemental area of the sensor surface is important in determining the optimum location of the sensor in a calibration setup. Reference [15] gives the expressions for determining the point variation of CF at the sensor surface.

**Fig. 12:**
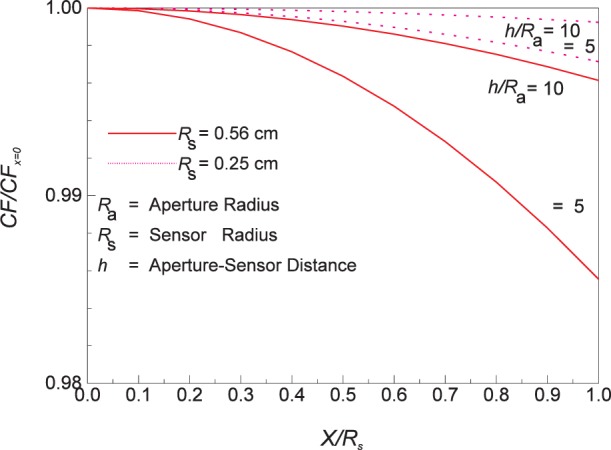
Plot variation of the configuration factor *CF* across the sensor radius for the NIST 25 mm variable temperature balckbody (VTBB).

Figure 12 shows the variation of *CF* across the sensor surface for the 25 mm VTBB for two sensors of 0.25 cm and 0.56 cm radius. The sensor radius of 0.25 cm corresponds to typical heat flux gages. For this case, the change in CF from the center to the edge of the sensor is less than 0.5 % for the sensor located at five times the blackbody aperture radius (*h*/*R*_a_ 5). This location corresponds to the sensor position where a heat flux level of about 100 kW/m^2^ can be obtained with the 25 mm VTBB. The 0.56 cm sensor radius corresponds to the aperture size of the transfer standard ECR. The drop off in CF in this case is about 1.5 % at the edge for the same location. This suggests that a correction to the heat flux value measured by the ECR may be required while transferring the calibration to the sensor. This is particularly important in the development of the absolute calibration technique when a large aperture sensor is located close to the radiating cavity aperture.

## 6. Future Plans

Further research to improve and standardize the present transfer technique of calibrating heat flux gauges is now in progress. To extend the transfer technique to flux levels up to 100 kW/m^2^, a suitable transfer standard, like an ECR, which has long term repeatability, will be procured. Design changes to reduce temperature gradients along the length of the 25 mm NIST cylindrical-tube VTBB will be investigated. Reduction of these gradients offers the potential of using the VTBB as an absolute as well as a transfer source.

It is also planned to develop in parallel an absolute calibration system of the type described in Ref. [12]. A 230 mm diameter spherical furnace with a 50 mm radiating aperture will be procured for this purpose. Temperatures of the furnace walls and of other radiating surfaces within view of the sensor will be measured in order to accurately calculate incident heat flux. Use of the ECR from the transfer technique will provide a check on the calculated flux at the aperture of the spherical blackbody.

Once both transfer and absolute calibration capabilities are in place, the intent is to reinforce confidence in NIST radiative calibrations through direct comparative studies between these two independent techniques.
